# Barriers and bottlenecks to implementation of WHO recommendations for prevention, detection, and treatment of postpartum hemorrhage: a mixed-methods triangulation study

**DOI:** 10.1186/s43058-026-00912-3

**Published:** 2026-06-10

**Authors:** Caitlin R. Williams, Sara Rushwan, Kristie-Marie Mammoliti, A. Metin Gülmezoglu, Lester Chinery, Arri Coomarasamy, Fernando Althabe, Mariana Widmer, Allisyn Moran, Uzma Syed, Olufemi T. Oladapo, Ioannis Gallos

**Affiliations:** 1 UNDP/UNFPA/UNICEF/WHO/World Bank Special Programme of Research, Development and Research Training in Human Reproduction, Department of Sexual, Reproductive, Maternal, Child, Adolescent Health and Ageing, WHO, Geneva, Switzerland; 2 Concept Foundation, Geneva, Switzerland; 3 Nossal Institute for Global Health, University of Melbourne, Melbourne, Australia; 4 Nuffield Department of Women’s & Reproductive Health, University of Oxford, Oxford, UK

**Keywords:** Postpartum hemorrhage, Implementation science, Health planning, Health services research, Resource-limited settings

## Abstract

**Background:**

The World Health Organization develops evidence-based recommendations for postpartum hemorrhage (PPH) prevention, detection, and treatment. However, persistently high PPH-related mortality suggests recommendations are poorly implemented, especially in low-resource settings. This study aimed to identify implementation bottlenecks to inform action.

**Methods:**

Drawing on public policy, implementation science, and health behavior, we constructed a multi-level framework to identify bottlenecks affecting recommendation uptake. This informed the development of a survey assessing which bottlenecks affected specific WHO recommendations. Surveys were completed by global stakeholders and by health care workers in three high-burden countries. We also analyzed direct observations of clinical practice from those countries to see how bottlenecks affected practice. An a priori interpretive threshold of 75% was selected to indicate a bottleneck was not a severe impediment to implementation.

**Results:**

Surveys from 102 global stakeholders and 246 health care workers, as well as 2039 direct observations of clinical practice revealed variation in adherence to WHO recommendations, with some through-lines. Oxytocin for PPH prevention and treatment was perceived to have national policy support (> 75% for inclusion in national guidelines and Essential Medicines Lists, regulatory approval, and support from national champions) but other uterotonics were not. Tranexamic acid for PPH treatment was found to be underutilized, likely due to compounding bottlenecks. Respondents reported extensive bottlenecks to refractory PPH interventions (< 75% across nearly all bottlenecks). The most substantial bottlenecks were availability/procurement of medicines and devices, affordability and quality of medicines and devices, presence of job aids, and availability of qualified staff. Observed practice generally aligned with stakeholder perceptions.

**Conclusions:**

National policy bottlenecks strongly mediated implementation in clinical practice. Policy change addressing gaps in commodity procurement and availability of trained clinicians is critical. Efforts addressing only one part of the implementation ecosystem are unlikely to drive transformative change.

**Supplementary Information:**

The online version contains supplementary material available at 10.1186/s43058-026-00912-3.

Contributions to the literature

- This paper synthesizes theoretical insights spanning the outer and inner settings of implementation to develop a consolidated multi-level theory of why implementation of evidence-based interventions stall.
- The empiric results show that bottlenecks in national policy hinder subsequent downstream implementation of WHO recommendations to prevent, detect, and treat postpartum hemorrhage. With most implementation strategies targeting the inner setting, our findings highlight the need to develop and test strategies for affecting change in the outer setting.
- The study offers one approach for using theory-derived implementation research to identify gaps where targeted interventions have catalytic potential to drive improvements in global maternal health.

## Background

Combating postpartum hemorrhage (PPH) is a key global maternal health priority. Obstetric hemorrhage is the leading cause of maternal death worldwide, accounting for an estimated 27% of maternal mortality, with PPH constituting the largest share of hemorrhage-related mortality [1]. In addition, PPH is an important contributor to long-term maternal morbidity, with far-reaching consequences including mental health conditions like post-traumatic stress disorder and postpartum depression [2, 3]. To aid health care workers in more effectively preventing and treating PPH, the World Health Organization (WHO) has issued several PPH guidelines, distilling research evidence into clinical and health systems recommendations for providing quality PPH care [4–12]. Yet these recommendations have not translated into the improvements in maternal health outcomes needed to meet Sustainable Development Goal (SDG target 3.1) [13]. There is a need to better understand why.

This study presents a novel theory-based approach to describe key steps on the pathway to implementation of global guidelines and assess how current efforts to translate WHO’s evidence-based PPH recommendations into routine practice have stalled. The results of this study can be used to inform global efforts to accelerate uptake of WHO guidelines, as well as address barriers and bottlenecks to implementation – particularly those that arise in the outer setting of implementation [14].

### Understanding barriers and bottlenecks to implementation

The need to understand why implementation does not go according to plan has spawned numerous fields of inquiry, among them implementation science, process evaluation, and policy implementation research. Within implementation science, barriers to implementation are defined as those determinant factors that can slow, stall, or derail the uptake or sustainment of an intervention [15]. “Barriers to implementation” is an overarching category that encompasses problems which may arise at various levels or stages within implementation. Efforts are underway to more precisely categorize the mechanisms by which barriers may impede implementation [16]. Yet thus far, these categorizations have remained largely descriptive with limited explanatory utility [17]. A different type of conceptual framing is needed to move from describing *what* barriers exist to explaining *why* and *how* barriers impede implementation.

Tanahashi’s model of bottlenecks to health service provision suggests that implementation may break down at discrete points in a service delivery process, which can be detected by comparing coverage measures drawn from adjacent levels of service delivery (e.g., coverage of a screening intervention versus coverage of a treatment intervention among individuals screened positive) [18]. Baker et al. extend this concept to posit that there are identifiable moments in an implementation pathway—implementation bottlenecks—when implementation becomes stuck [19]. For example, efforts to bring a new medical device to market worldwide may slow at the regulatory approval stage, especially in countries with poorly resourced regulatory agencies [20]. Where “barriers to implementation” are theory-agnostic, “bottlenecks to implementation” imply a presupposed mechanism by which implementation should occur, and the existence of an impediment thwarting a specific step in that mechanism. Thus, identifying bottlenecks to implementation can help determine how to unstick efforts to translate evidence to policy and practice and get life-saving interventions into the field [21].

Despite the utility of mapping bottlenecks to implementation, this step is often overlooked. One reason is that identifying bottlenecks to implementation can be costly and time-consuming. Traditional efforts may entail conducting facility audits, detailed needs assessments, or in-depth interviews with key stakeholders [22]. These approaches generate useful information but can be limited by the perspectives included and excluded, since different stakeholder groups may have varying understandings of where and why implementation has stalled. Developing a comprehensive picture of the factors influencing uptake of evidence-based interventions often requires engaging multiple stakeholder groups, thus increasing cost and logistical complexity. Further, traditional approaches are typically atheoretical and thus may miss important aspects of implementation. Innovative methods are needed that systematize data collection on implementation bottlenecks while reducing burden on researchers, policymakers, and others interested in gathering and using such information. Our study offers one such approach.

## Methods

This mixed-methods triangulation study [23] used three sources of data—1) global stakeholder responses to a theory-derived survey on potential bottlenecks to implementation, 2) direct observations of clinical practice conducted in three countries (Nigeria, Pakistan, and Tanzania), and 3) observed health care workers’ responses to a theory-derived survey of potential bottlenecks to implementation—to identify specific factors potentially hindering implementation of evidence-based recommendations for PPH prevention and management. More specifically, data were used to detect evidence-based recommendations that were not well-implemented in the field and to identify common recommendations that had proved challenging to implement across all three settings. Though implementation is often described as highly contextualized [24], common challenges may suggest a need for global cooperation to address supranational barriers (e.g., conflicting normative guidance, regulatory ambiguity, procurement and supply chain issues). To organize and structure data collection and integration, a consolidated conceptual framework was developed, drawing from the existing theoretical implementation science literature.

### Consolidated conceptual framework

The Consolidated Framework for Implementation Research (CFIR) suggests that clinical interventions are provided within a care ecosystem that has both an inner setting (within-facility context) and an outer setting (policy, economy, society) [14]. For a new intervention to be administered at bedside, there is a series of changes that must first occur in the outer and inner settings in which service delivery occurs. For example, the intervention needs to have been approved by national regulatory bodies, integrated into domestic policy and guidance, embedded into pre-/in-service training, and actively encouraged within the facility (through, for example, inclusion in clinical protocols, reinforcement by supportive supervision, and active engagement of facility leadership). Without undertaking these steps to transform the domestic care ecosystem into an enabling environment, the intervention cannot be effectively delivered to patients. Yet despite a wealth of implementation-related theorizing, no existing implementation science theory, model, or framework currently covers the steps involved in restructuring the outer and inner settings to produce an enabling environment [25, 26]. Creating an enabling environment requires multiple instances of policy transfer and knowledge translation across different levels and stakeholder groups, each with their own mental models and operating procedures [27–29]. Academics have traditionally partitioned theorizing around these levels and groups, with discrete epistemic communities (e.g., policy implementation, organizational theory, health behavior) developing different bodies of theoretical work (e.g., top-down implementation, organizational readiness, theory of planned behavior) [30–32]. Yet effectively moving global recommendations into local practice requires integrating theoretical constructs from across these disparate academic traditions into a single consolidated framework.

**Fig. 1:**
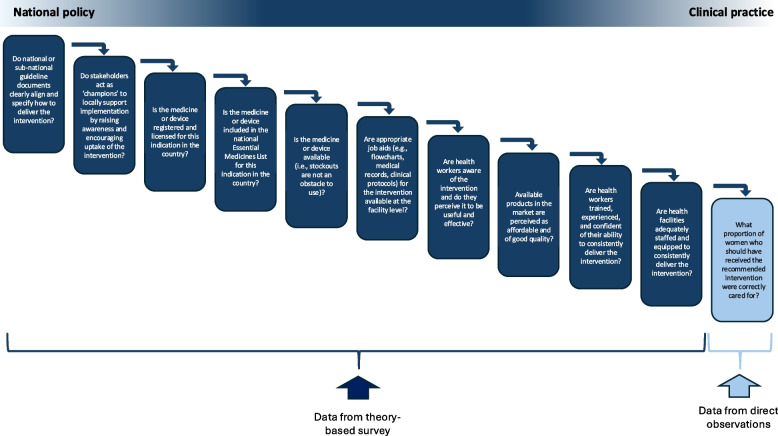
Conceptual model of barriers and bottlenecks to national implementation of WHO-recommended interventions to prevent, detect, and treat postpartum hemorrhage

Over the past decade, UNICEF has developed and refined an approach for mapping bottlenecks to implementation that extends the Tanahashi model to show how bottlenecks to implementation can be characterized as determinants of supply, demand, or quality [33]. That approach has been used by UNICEF as a first step to improve complex health system performance within countries [21]. Our analysis builds from the UNICEF approach to conceptually map the supply, demand, and quality considerations of implementing WHO PPH recommendations from policy to clinical practice (i.e., transferring from global to national to sub-national to facility) in a single consolidated framework. The consolidated framework draws on theorizing in policy implementation and implementation science, as well as global health practice-based evidence and WHO normative work. Using a top-down conceptualization of policy implementation [30] and following from Baker et al.’s operationalization of Tanahashi’s model [19], the process of moving from evidence-based recommendations to clinical practice was envisioned as a unidirectional implementation pathway in which addressing bottlenecks in earlier steps created more enabling environments for subsequent steps. Discrete steps required to embed global guidelines into national policy were identified, using concepts from the literature on policy transfer [27–29]. Next, steps needed to translate national policy into clinical practice were identified, building from the literature on implementation research and practice [34–37]. Finally, each of these steps was mapped against the WHO building blocks of health systems [38], to ensure that no elements had been overlooked. Given the critical role of commodities such as uterotonics, tranexamic acid, and devices for refractory PPH in PPH prevention and management, special attention was paid to tracing the steps involved in ensuring access to quality commodities at the bedside. The integrated conceptual framework is provided in Fig. 1.

### Survey of global stakeholders

To better understand the upstream factors contributing to poor implementation at the point of care, a targeted online survey was conducted to capture global stakeholders’ perceptions of the specific steps in the cascade at which implementation of PPH recommendations was stalled. Within policy research, in-depth interviews with key informants are commonly used to efficiently gather insight into policy-related processes, including policy transfer [39]. However, managing large-scale qualitative data analysis can be time-consuming and resource intensive. Theory-derived surveys can offer a more efficient approach to large-scale data collection [40]. The conceptual framework was used to inform the development of the survey questions, with one question developed for each step in the implementation pathway. Recognizing that different recommendations might face different challenges to implementation, the survey was structured so that respondents answered the questions in the pathway for one evidence-based recommendation at a time. The full set of questions was posed for recommendations regarding specific PPH commodities (medicines and devices). A subset of questions was posed for recommendations on clinical or health system interventions, where the items on procurement and regulatory approval were not applicable. A blank copy of the survey questionnaire is provided in Supplementary file S1.

The survey was shared with 105 global stakeholders for an initial round of prioritization. These stakeholders were drawn from Ministries of Health, women and women’s groups, implementers (including non-governmental organizations and civil society organizations), professional associations, guideline developers, the research community, international donors, and the private sector – all acting in an individual capacity and not representing any specific organization. The composition and selection of this global stakeholder group have been described previously and were not modified for the present study [41]. Participants were sent a unique link to an online survey (via SurveyMonkey). Submission of the survey indicated consent to participate. The survey was open for five weeks (25 January 2023–1 March 2023).

Questions were posed by WHO PPH recommendation. Participants were asked to report the extent to which they agreed that the statement posed was true (e.g., “Medicine or device is registered and licensed for this/these indication/s in the country”). Responses were on a 5-point Likert scale, ranging from “Strong disagree” to “Strongly agree.” To minimize forced responses and reduce noise, respondents were able to select “Don’t know” for items they felt unable to assess. At the analysis stage, aggregate scores were calculated as simple means of item responses, using a linear transformation in which 0 corresponded to “Strongly disagree” and 1 to “Strongly agree”; “Don’t know” responses were excluded from analysis by design. Aggregate scores were visualized using heatmaps to facilitate identification of potential bottlenecks. To aid interpretation, a score of 75% was used as an a priori interpretive threshold to distinguish pathway steps for which, on average, respondents indicated agreement that the step was in place from those falling below this level. Under the linear transformation used, a score of 0.75 corresponds to the Likert response option “Agree.” This threshold was selected for interpretability and consistency with the scale anchors rather than as a statistical decision rule. Respondents were also invited to provide explanatory comments on each intervention via open-ended questions, including descriptions of barriers not captured by the structured items. These comments were reviewed, grouped by theme, and summarized.

### Direct observations of clinical practice

To test the face validity of the findings of the global survey, direct observations of health care providers’ clinical practice in three countries with high burden of PPH-related morbidity and mortality (Nigeria, Pakistan, and Tanzania) were reviewed and triangulated with global participants’ survey responses. Direct observations were drawn from a nested study within a large implementation trial for the early detection and treatment of postpartum hemorrhage and a pre-post evaluation of an effort to implement the same complex intervention [42–44]. For the purposes of the current study, the direct observations constitute a convenience sample.

Observations were drawn from 19 facilities in Nigeria, 6 facilities in Tanzania, and 2 facilities in Pakistan. In Nigeria and Tanzania, observations were conducted at each facility over a two-week period between August 2022 and December 2022 (prior to the start of implementation of the early detection and treatment intervention), covering 24 hours Monday to Sunday to reduce risk of selection bias and gain insights into the care provided across all shifts during the day, night, weekdays, and weekends. In Pakistan, observations were conducted over a 3–4-day period in both sites between January and February 2023 (prior to the start of implementation of the early detection and treatment intervention). Observation periods varied across countries due to logistical and contextual constraints; however, observations in all settings were designed to capture routine care processes rather than to estimate frequencies or rates. Research staff observed in real time, as health care workers employed by the facility attended vaginal births, administered preventive interventions, conducted direct observations, and managed any PPHs that occurred. Data were collected using a purpose-designed structured guide. Observations were conducted in the labor ward. If a woman was transferred to theatre for escalated treatments, some data on refractory care was obtained from the main study database, which was entered by the research staff after confirmation of treatment by the treating clinician.

### Survey of health care workers

For completion, health care workers whose care provision was observed were invited to fill in the same survey as global stakeholders, to assess generalizability of the global findings to their contexts. Scores were tabulated, aggregated, and analyzed in the same way as for the global stakeholder survey, but analyses were conducted independently for each country.

All analyses were descriptive in nature and intended to identify patterns of perceived implementation bottlenecks rather than to formally test hypotheses or estimate population-level effects. Given the purposive sampling strategy and the diagnostic aim of the study, inferential statistical testing was not performed.

## Results

### Global stakeholder survey

**Fig. 2:**
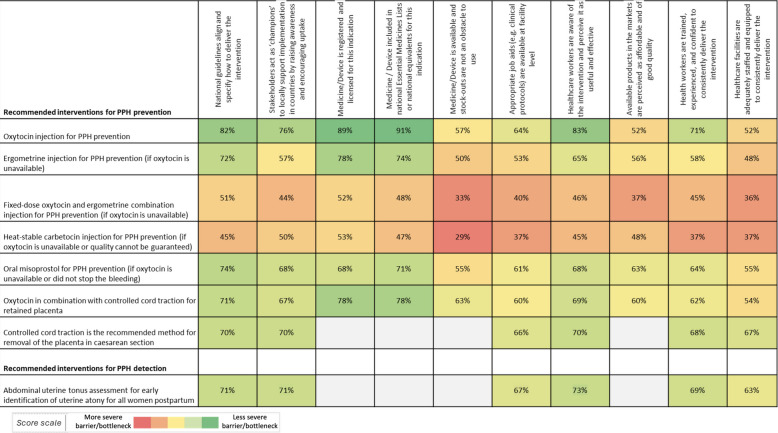
Global stakeholder perspectives on barriers and bottlenecks to national implementation of WHO recommendations for the prevention and detection of postpartum hemorrhage

**Fig. 3:**
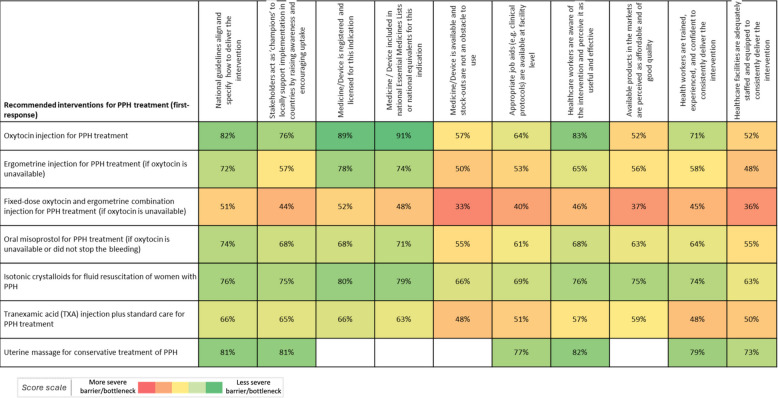
Global stakeholder perspectives on barriers and bottlenecks to national implementation of WHO recommendations for the first-response treatment of postpartum hemorrhage

**Fig. 4:**
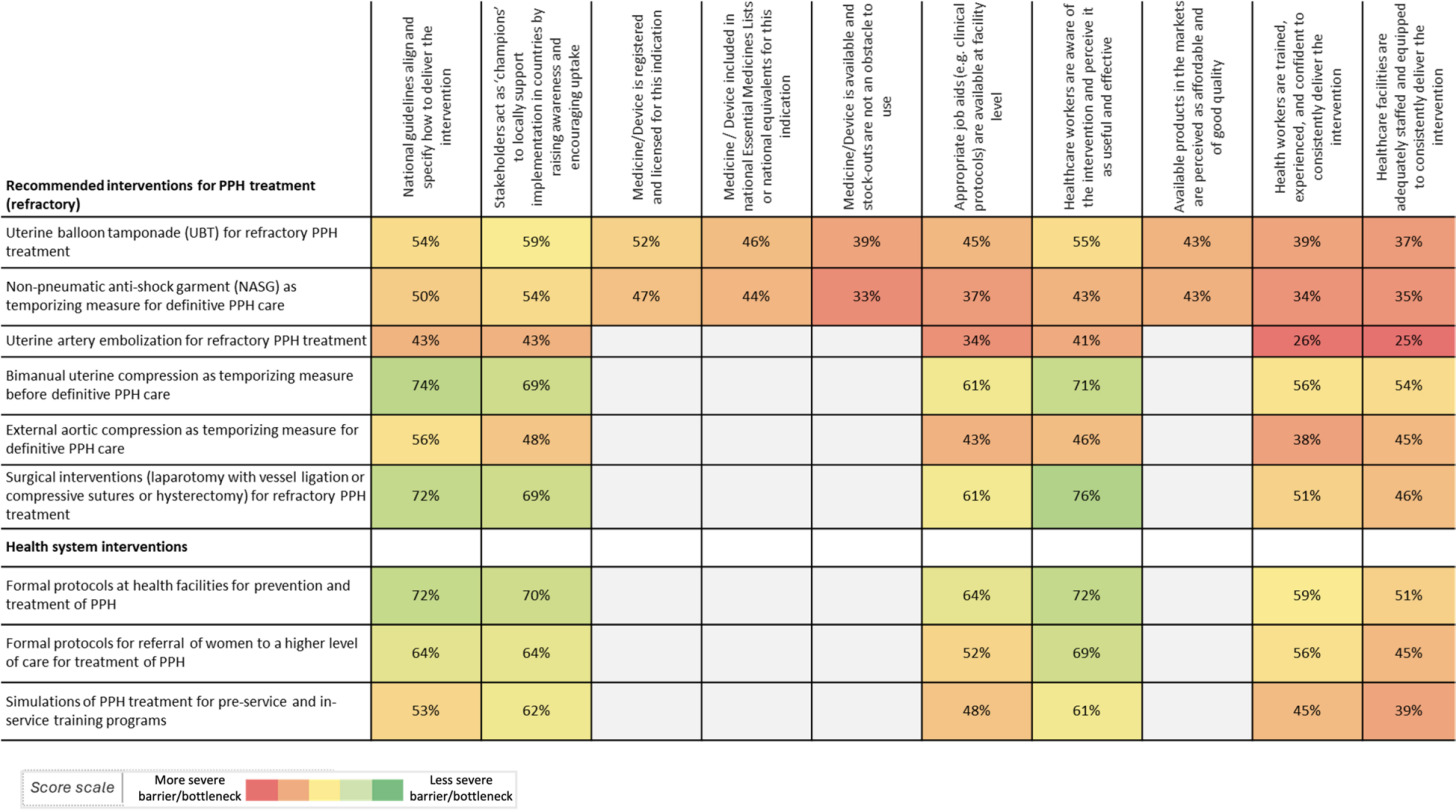
Global stakeholder perspectives on barriers and bottlenecks to national implementation of WHO recommendations for the treatment of refractory postpartum hemorrhage and health system interventions

The global survey was sent to 105 global stakeholders, of whom 102 consented and responded (response rate: 97.1%). Aggregate scores are plotted on the heatmap in Figs. 2, 3 and 4. “Don’t know” responses were rare for widely disseminated recommendations (e.g., oxytocin for PPH prevention) and more frequent for more technical clinical interventions, particularly among non-clinical respondents, reflecting differences in role relevance and exposure. This pattern was consistent with the study’s intent to capture informed perceptions of implementation bottlenecks rather than uncertainty regarding the recommendations themselves. The overall pattern of identified bottlenecks was robust to alternative interpretive thresholds (70% and 80%), with differences limited to a small number of items near the cutoff. Certain interventions were perceived as broadly facing bottlenecks to implementation across the entire implementation pathway, while others were perceived to be reasonably well-implemented across the pathway. Four steps in the pathway (availability/procurement of medicines and devices; affordability and quality of medicines and devices; presence of job aids in the facility; and availability of qualified staff) consistently posed more substantial bottlenecks to implementation than others, regardless of the intervention. Stakeholder comments in the open-ended questions helped to contextualize the aggregate scores and showcase the existence of other important barriers.

Oxytocin use was generally perceived to face few bottlenecks to implementation for both PPH prevention and treatment, whereas stakeholders reported bottlenecks to implementation across the entire implementation pathway for other uterotonics recommended for PPH prevention (or treatment if oxytocin was unavailable). In open-ended comments, participants indicated that implementation was sometimes stalled due to barriers not captured by the pathway. For example, ergometrine was perceived to be underused because of concerns about side-effects; fixed-dose oxytocin-ergometrine was perceived to be “out of fashion”; and heat-stable carbetocin was reported to be still relatively new, unknown, and costly as compared to other prophylactic uterotonic options. Global stakeholders perceived oral misoprostol for PPH prevention and treatment, tranexamic acid for PPH treatment, prophylactic oxytocin with controlled cord traction, and isotonic crystalloids for fluid resuscitation as facing fewer challenges as compared to other interventions.

In contrast, stakeholders perceived device-based and surgical interventions for refractory PPH as facing extensive barriers and bottlenecks to implementation. Stakeholders commented that uterine balloon tamponade (UBT) and the non-pneumatic anti-shock garment (NASG) were rarely used in practice because the devices were largely unavailable and/or perceived to be unaffordable, were not included in facility-level protocols, and generally had not been well-promoted or championed in-country. Uterine artery embolization was perceived to be available only in high-level tertiary facilities with specialists, and even then, as often difficult to access. Similar points were raised for other surgical procedures, though to a lesser extent. Lack of available staff and staff time were the primary points raised in global stakeholder comments with respect to clinical interventions such as uterine tone assessment for identification of women with postpartum uterine atony, controlled cord traction combined with oxytocin for removal of retained placenta, and external aortic compression as a temporizing measure.

Global stakeholder perceptions on health system interventions also varied by intervention, with simulation drills having the greatest number of perceived barriers to implementation. In open-ended comments, global stakeholders reported that simulation drills were particularly hindered by lack of trained personnel to conduct drills and understaffing of frontline health care workers. In addition, simulation drills were reported to be not well-promoted domestically.

### Direct observations of clinical practice and survey of health care workers

A total of 1352 direct observations were conducted in Nigeria (77 cases of PPH diagnosed and treated). In Tanzania, 649 direct observations were conducted (72 cases of PPH diagnosed and treated) and in Pakistan, 38 direct observations were conducted (7 cases on PPH diagnosed and treated). Direct observation results are reported in Table 1. In addition, a total of 246 health care workers responded to the theory-based survey (31 in Nigeria, 126 in Pakistan, and 50 in Tanzania). Heatmaps of health care workers’ aggregate survey scores for each of the countries are provided in Supplementary file S2.

**Table 1:** Administration of recommended interventions for PPH prevention, detection, and treatment during direct observations in Nigeria, Pakistan, and Tanzania

	**Nigeria**		**Pakistan**		**Tanzania**	
**Recommended interventions for PPH prevention**	**%**	**n** (*N*=1352 observations in 19 facilities)	**%**	**n** (*N*=38 observations in 2 facilities)	**%**	**n** (*N*=649 observations in 6 facilities)
Oxytocin injection for PPH prevention	99.7%	1348	100.0%	38	100.0%	649
*Oxytocin infusion also administered*	*3.4%*	*46*	*0.0%*	*0*	*5.5%*	*36*
Ergometrine injection for PPH prevention (if oxytocin is unavailable or quality cannot be guaranteed)	0.4%	6	0.0%	0	0.0%	0
Fixed-dose oxytocin and ergometrine combination injection for PPH prevention (if oxytocin is unavailable or quality cannot be guaranteed)	0.0%	0	0.0%	0	0.0%	0
Heat-stable carbetocin injection for PPH prevention (if oxytocin is unavailable or quality cannot be guaranteed)	0.7%	9	0.0%	0	0.0%	0
Misoprostol for PPH prevention (if oxytocin is unavailable or quality cannot be guaranteed)	61.5%	832	78.9%	30	2.0%	13
* Oral or sublingual* * Per Rectal* * Both Oral or Sublingual and Per Rectal*	*13.8% 51.8% 4.0%*	*186* *700* *54*	*2.6* *78.9% * *2.6%*	*1* *30* *1*	*0.2%* *1.8% * *0.0%*	*1* *12* *0*
**Recommended interventions for PPH detection**	**%**	**n** (*N*=1352 observations in 19 facilities)	**%**	**n** (*N*=38 observations in 2 facilities)	**%**	**n** (*N*=649 observations in 6 facilities)
Abdominal uterine tonus assessment for early identification of uterine atony for all women postpartum during direct observations*	64.4%	898	100.0% %	38	73.0%	474
**Recommended interventions for PPH treatment (first-response)**	**%**	**n** (*N*=77 PPHs diagnosed)	**%**	**n** (*N*=7 PPHs diagnosed)	**%**	**n** (*N*=72 PPHs diagnosed)
Oxytocin injection for PPH treatment	70.1%	54	100.0%	7	98.6%	71
Ergometrine injection for PPH treatment (if oxytocin is unavailable or quality cannot be guaranteed)	0.0%	0	0.0%	0	0.0%	0
Fixed-dose oxytocin and ergometrine combination injection for PPH prevention (if oxytocin is unavailable or quality cannot be guaranteed)	0.0%	0	0.0%	0	0.0%	0
Misoprostol for PPH treatment (if oxytocin is unavailable, quality cannot be guaranteed or did not stop the bleeding)	41.6%	32	28.6%	2	36.1%	26
* Oral or sublingual* * Per Rectal* * Both Oral or Sublingual and Per Rectal*	*15.6%* *29.9%* *3.9%*	*12* *23* *3*	*0.0%* *28.6% * *0.0%*	*0* *2* *0*	*1.4% * *34.7%* *0.0%*	*1* *25* *0*
Tranexamic acid (TXA) injection for PPH treatment	27.3%^‡^	21^‡^	100.0%^‡^	7^‡^	48.6%^‡^	35^‡^
Isotonic crystalloids for fluid resuscitation of women with PPH	62.3%	48	100.0%	7	79.2%	57
Oxytocin in combination with controlled cord traction for retained placenta	-	1	-	None occurred	-	3
Uterine massage for conservative treatment of PPH	92.2%	71/77	100.0%	7/7	91.7%	66
**Recommended interventions for PPH treatment (refractory)**	**%**	**n**	**%**	**n**	**%**	**n**
Uterine balloon tamponade (UBT) for refractory PPH treatment	-	None occurred	-	None occurred	-	2
Non-pneumatic anti-shock garment (NASG) as temporizing measure for definitive PPH care	-	1	-	None occurred	-	None occurred
Uterine artery embolization for refractory PPH treatment	-	None occurred	-	None occurred	-	None occurred
Bimanual uterine compression as temporizing measure	-	4	-	None occurred	-	26
External aortic compression as temporizing measure for definitive PPH care	-	None occurred	-	None occurred	-	None occurred
Surgical interventions (laparotomy with vessel ligation or compressive sutures or hysterectomy) for refractory PPH treatment	-	1	-	None occurred	-	None occurred
**Health system interventions**	**%**	**n** (*N*=19 facilities)	**%**	**n** (*N*=2 facilities)	**%**	**n** (*N*=6 facilities)
Formal protocols at health facilities for prevention and treatment of PPH	Yes: 42.1% No: 47.4% Unknown: 10.5%	8 9 2	Yes:100.0% No: 0.0 % Unknown: 0.0%	2 0 0	Yes: 83.3 % No: 16.7 % Unknown: 0.0%	5 1 0
Formal protocols for referral of women to a higher level of care for treatment of PPH	Yes: 0.0% No: 100.0%	0 19	Yes: 50.0% No: 50.0%	1 1	Yes: 0.0% No: 100.0%	0 6
Simulations of PPH treatment for pre-service and in-service training programs	Yes: 5.3% No: 94.7%	1 18	Yes: 100.0% No: 0.0%	2 0	Yes: 83.3% No: 16.7%	5 6

* Numerator: number of times uterine tone checked for 1^st^ direct observation; Denominator: number of discrete HCP observations (women)

^‡^ Variations occur in standard care

Oxytocin was widely observed in use for PPH prevention (Nigeria: 99.7% of observed births; Pakistan: 100.0%; Tanzania: 100.0%). Although oral misoprostol was only recommended for prevention when oxytocin was unavailable or the quality could not be assured, a substantial proportion of women in Nigeria and Pakistan were administered both oxytocin and oral misoprostol for PPH prevention (Nigeria: 61.5% of observed births; Pakistan: 78.9%; Tanzania: 2.0%). In Nigeria, the aggregate health care worker survey score indicated that “Available products in the markets are perceived as affordable and of good quality” was a barrier to implementation of oxytocin but not to that of oral misoprostol. In direct observations, ergometrine, fixed-dose oxytocin and ergometrine combination, and heat-stable carbetocin were rarely used for prevention (fewer than 1% of observed births in all three countries). In Pakistan and Tanzania, health care worker aggregate survey scores indicated extensive perceived barriers to implementation of recommendations of second-line uterotonics.

Postpartum uterine tone was assessed to help detect PPH in most cases (Nigeria: 64.4% of observed births; Pakistan: 100.0%; Tanzania: 73.0%). In the health care worker aggregate survey responses, adequate staffing was the single reported barrier or bottleneck to implementation of this recommendation in Nigeria and Tanzania.

When it came to PPH treatment, oxytocin was widely observed in use (Nigeria: 78.1% of observed PPH cases; Pakistan: 100.00%; Tanzania: 98.6%). Oral misoprostol was used extensively for treatment (Nigeria: 41.6% of observed PPH cases; Pakistan: 28.6%; Tanzania: 36.1%). Ergometrine and fixed-dose oxytocin and ergometrine combination were rarely used for PPH treatment (fewer than 1% of observed PPH cases in all three countries). Although tranexamic acid is recommended as a first-response treatment for PPH, use varied widely across settings (Nigeria: 27.3% of observed PPH cases; Pakistan: 100.0%; Tanzania: 48.6%). In Pakistan, health care workers did not report perceived barriers to implementation of tranexamic acid. In Nigeria and Tanzania, however, aggregate health care worker survey responses indicated that there were multiple barriers to implementation, ranging from availability challenges to poor health care worker awareness of its utility through adequate training and support for use in practice. Use of isotonic crystalloids for fluid resuscitation was also context-dependent (Nigeria: 62.3% of observed PPH cases; Pakistan: 100.0%; Tanzania: 79.2%), though health care worker survey responses did not identify any specific barriers or bottlenecks to implementation. Uterine massage for conservative treatment of PPH was conducted nearly universally (Nigeria: 92.2% of observed PPH cases; Pakistan: 100.0%; Tanzania: 91.7%).

Use of recommended interventions for refractory PPH were rarely observed, though this is primarily reflective of the small numbers of refractory PPH cases observed. However, health care worker aggregate survey responses indicated the existence of substantial barriers and bottlenecks to implementation across the entire pathway for recommended interventions to treat refractory PPH.

Notable findings among health system interventions include the lack of referral protocols for transferring women with refractory PPH to higher levels of care (Nigeria: 0% of facilities had a protocol; Pakistan: 50%; Tanzania: 0%) and the limited use of simulation drills in Nigeria (only 1 of the 19 participating facilities reported using simulation drills). Interestingly, only health care worker aggregate survey responses in Nigeria indicated that there were bottlenecks to implementation of these kinds of health system interventions. Health care workers in the other two countries did not perceive any bottlenecks to implementation of these recommendations.

## Discussion

This study sought to identify WHO evidence-based recommendations for PPH prevention and treatment whose implementation are stalled and characterize some of the bottlenecks to implementation, using a combination of real-time direct observations of clinical practice and theory-derived surveys of global stakeholders’ and local health care workers’ perceptions. Among uterotonics, only oxytocin and misoprostol seemed to be readily available, but there are known quality concerns with oxytocin and misoprostol in many low-resource settings [45–49]. Health care workers’ awareness of oxytocin quality concerns may have led them to compensate with additional oxytocin or simultaneous use of misoprostol, as observed in this study. However, it is unknown how much additional medication may be needed to compensate for poor quality, and such usages of PPH commodities may have other negative consequences. Recommended clinical maneuvers for PPH detection and treatment (uterine tone assessment and uterine massage) were observed to be widely used, likely due to the limited resources required (i.e., no commodities needed). Tranexamic acid for PPH treatment was found to be underutilized, likely due to the compounding effect of the multiple bottlenecks to implementation evidenced by this study. Some recommended interventions for treating refractory PPH were not available to health care workers, potentially contributing to underutilization and the desire to create improvised devices [50]. Health system interventions like formal facility protocols for PPH management and referral and simulation training for preparedness were found to be substantially underutilized. Instituting these interventions is perhaps easier and faster to accomplish than ensuring other procurement-dependent interventions are consistently available to health care workers.

Global stakeholders and observed health care workers reported more challenges to implementation at lower-level steps in the conceptual framework (e.g., provider practice) than at higher-level steps (e.g., national policy) for all interventions. Interventions that stakeholders reported as facing substantial barriers at higher-level steps in the pathway had particularly low rates of utilization in direct observations. This suggests that higher-level challenges to implementation may create rate-limiting constraints that hinder implementation at lower levels, contributing to certain interventions being plagued by poor implementation across the pathway.

Findings from direct observations support the premise that implementation is context specific. These data show that observed facilities in Nigeria were less aligned with global evidence-based recommendations than those in Tanzania and Pakistan. Accordingly, there may be more bottlenecks to implementation in Nigeria or the bottlenecks may be more entrenched. These findings align with global literature suggesting that efforts to improve uptake of evidence-based interventions should begin with an exploration stage that encompasses local assessment of bottlenecks, barriers, and facilitators of/to implementation (implementation determinants) [21, 51, 52].

Strengths of this study include the use of a theory-derived approach to measuring bottlenecks to implementation, the use of data triangulation to allow for comparison of global stakeholder perceptions to facility-level practices, and the high response rate to the stakeholder survey. Further, the mixed-methods stakeholder survey illustrated the perils of relying solely on quantitative data to map barriers and bottlenecks to implementation. The open-ended stakeholder comments revealed qualitative differences between the implementation challenges facing ergometrine and heat-stable carbetocin, for example, which would have been obscured with a solely quantitative survey. In this way, the use of multiple, complementary data sources, theories, and analytical approaches strengthened the overall study and utility of the findings to the field.

However, the study also has limitations. First, it is possible that the implementation pathway is missing key steps, obfuscating understanding of potentially important barriers or bottlenecks to implementation. Second, although we used a 75% threshold aligned with the ‘Agree’ anchor to structure interpretation, this value is inherently interpretive. Importantly, all item-level aggregate scores are reported, allowing readers to assess how alternative thresholds would affect classification, particularly for items near the cutoff. As the study was not designed to support probabilistic inference, we did not assess statistical significance of differences between interventions or countries; instead, credibility of findings rests on consistency of patterns across multiple data sources. Third, it is likely that these results understate the extent to which evidence-based PPH recommendations are underutilized in the field. Although direct observations were conducted in facilities not yet implementing an intervention, by nature of participating in a multi-country trial, these facilities were likely to have better than average implementation of evidence-based recommendations (given proximity to/involvement in global research networks and location and type of facility). In addition, direct observation of clinical care may have influenced provider behavior (Hawthorne effect), such that observed practices represent best-case scenarios even within these facilities. Together, these factors suggest that the true extent of implementation gaps in typical real-world settings may be greater than observed here. As the findings are intended to be illustrative rather than generalizable, this limitation does not diminish their value for identifying relative patterns of implementation bottlenecks. Finally, reflecting existing WHO PPH recommendations, this study focused primarily on interventions intended to be delivered within health facilities. These findings may have limited relevance for the many PPHs that occur in communities. More work may be needed to understand the kinds of community-level interventions that may be useful to prevent, detect, and treat PPH.

This study makes two primary contributions to the literature. The first is specific to PPH. By systematically mapping barriers to the implementation of evidence-based PPH recommendations, this study facilitates priority-setting within global maternal health. The global stakeholder survey identified four bottlenecks to implementation that were largely intervention-agnostic: availability/procurement of medicines and devices; affordability and quality of medicines and devices; presence of job aids in the facility; and availability of qualified staff. In addition, lack of supportive national policy (e.g., inclusion in national guidelines and Essential Medicines Lists) was identified as a key precipitating bottleneck – if national policy was not supportive, it was very difficult for the intervention to be utilized. During the WHO-convened Global Summit on Postpartum Haemorrhage held in Dubai, United Arab Emirates in March 2023, stakeholders further underscored the importance of addressing these common bottlenecks across contexts. Consequently, these bottlenecks have been prioritized for global action within the Implementation strategic area of the *WHO Roadmap to Combat Postpartum Haemorrhage, 2023–2030* [41]. Specifically, the first three categories of priority actions are: building clear national health policy and strengthening national and subnational leadership, improving availability and access to quality-assured and affordable PPH commodities at all levels of the health system, and ensuring availability of competent health care workers to deliver relevant good-quality facility-based services and commodities.

In addition to the topic-specific contribution, this study makes a methodological contribution to implementation science by demonstrating how theory can be used to organize and structure empiric studies of implementation. The theoretical landscape in implementation science is currently dominated by determinant frameworks and process models, with fewer explanatory theories [24, 53, 54]. In recent years, there have been calls for developing more theory-based approaches to implementation, but little guidance on how to do so [17, 55]. This paper offers one approach, using theoretical *bricolage* (syncretic integration of theories from disparate academic traditions [56, 57]) to develop a fit-for-purpose mid-range theory of pre-requisites to implementation that could then be used to identify barriers and bottlenecks. This mid-range theory intentionally linked different levels of the health system to illustrate one way in which the outer setting of implementation (e.g., policy and regulatory spaces) can shape the enabling environment for implementation within the inner setting (e.g., health facilities). This approach provides one example of how implementation-focused researchers might further integrate implementation science with health systems research and policy implementation to create more cohesive theorizing and research [58, 59].

## Conclusions

This study identified several critical implementation bottlenecks hindering further improvements in PPH-related morbidity and mortality. Notably, implementation bottlenecks occurring within the national policy space had far-reaching implications and contributed to compounding failure to implement evidence-based practices at lower-levels in the health system. In contexts where policy presents a barrier to implementation (for example, because midwives are not legally authorized to offer certain interventions), urgent actions to address outdated policies and create enabling environments for implementation are needed. Health care workers and facility leaders are important agents for change, but results will be underwhelming if efforts are limited to the implementation inner setting (e.g., health facilities) alone. Rather, systems approaches are needed.

As trusted professionals, health care workers are well-positioned to advocate with policymakers for the need for supportive policy environments that allow them to use evidence-based and life-saving interventions in their clinical practice. Securing high-level leadership from policymakers can set the stage for transformative change. Consequently, evidence on the most effective advocacy strategies for engaging these leaders is needed [60].

In addition, more research is needed on the most effective implementation strategies for affecting change at the nexus between inner and outer settings. Whole-of-system theorizing and approaches to implementation research could help to drive this dimension of the field and address the top-priority implementation-related research question generated by participants at the Global Summit on Postpartum Haemorrhage [60]. Strategically embedding implementation research into future implementation efforts could reap a double dividend of generating much-needed insights while reducing PPH-related death and disability.

## Supplementary Information

Supplementary Material 1.

Supplementary Material 2.

## Data Availability

The survey datasets used and analyzed in the study are available from the corresponding author on reasonable request. The data that support findings from the clinical observations were provided for the purposes of this analysis by the authors of the E-MOTIVE Trial. Data requests for that dataset will be considered by the E-MOTIVE trial management group upon request to the Principal Investigator: arri.coomarasamy@wrh.ox.ac.uk.

## Abbreviations

CFIR: Consolidated Framework for Implementation Research
NASG: Non-pneumatic anti-shock garment
SDG: Sustainable Development Goal
PPH: Postpartum hemorrhage
UBT: Uterine balloon tamponade
WHO: World Health Organization
