# Supplementary material for: Barriers and bottlenecks to implementation of WHO recommendations for prevention, detection, and treatment of postpartum hemorrhage: a mixed-methods triangulation study
Source: Implement Sci Commun. 2026 Jun 10;7:153. doi: 10.1186/s43058-026-00912-3 (PMC13483687; doi:10.1186/s43058-026-00912-3)
Supplement: Supplementary file 1 [file 43058_2026_912_MOESM1_ESM.pdf]

WHO Survey on Barriers to Implementation of PPH Interventions

Introduction

Background and rationale

Postpartum haemorrhage (PPH) is the leading cause of maternal mortality worldwide. Despite the shared ambition to end preventable maternal deaths by 2030, several countries are not on track to meet their SDG maternal mortality targets. With the 2030 deadline for the SDGs fast approaching, WHO is mobilizing the international community to develop concrete plans to address gaps relating to the leading causes of maternal death. As part of this global effort, WHO is conducting a survey to identify key implementation bottlenecks to WHO-recommended PPH interventions that could hinder improvement in quality and outcomes of PPH care.

Aim of this exercise

WHO is seeking input from key stakeholders to gain insights into barriers to implementation of current WHO-recommended PPH interventions. This will help to provide a better understanding of specific implementation gaps that need to be addressed at the national and international level. The interventions have been drawn directly from the WHO recommendations. The questions listed for each intervention are designed to target domains along the implementation pathway.

Through your participation in this survey, WHO will capture information on barriers to implementation of WHO-recommended interventions at the national and international levels. This will inform the development of the implementation agenda for 2023 to 2030.

Please note that this survey is for you, as the target recipient, only and should not be forwarded to a colleague to submit. Specific instructions on how to provide your input are to follow.

World Health Organization

human reproduction programme hrp
research for impact
UNDP · UNFPA · UNICEF · WHO · WORLD BANK

WHO Survey on Barriers to Implementation of PPH Interventions

Respondent demographic information

Name

[ ]

Email

[ ]

* Expertise (select all that apply)

- [ ] Research
- [ ] Clinical care
- [ ] Development of novel medications/devices
- [ ] Programme implementation
- [ ] Policy making
- [ ] Financing

* Place of work (country)

[ ]

* Organization type

- ( ) Clinical/Health care facility
- ( ) Academic/Research
- ( ) Ministry of Health
- ( ) Non-Governmental Organization
- ( ) Professional association
- ( ) UN agencies and their partnerships
- ( ) Donor
- ( ) Industry and private sector
- ( ) Other (please specify)

[ ]

Background (cont.)

* To which sector does the health care facility where you work pertain?

- Public
- Private
- Both
- Other (please specify)

WHO Survey on Barriers to Implementation of PPH Interventions

The scope of this implementation exercise

The aim of this survey is to identify implementation gaps against implementation pre-requisites for WHO-recommended interventions.

The survey has been divided into two tracks:

1. **Medicines/Devices**
2. **Clinical and health system interventions**

There are ten (10) interventions listed for each track. Please submit your assessment for both tracks.

Please review and score your level of agreement depending on your best available knowledge, expertise, and experience on the extent to which the listed pre-requisite for each intervention is satisfied, globally or for a specific country.

* Are you responding to this survey from a global perspective, or from the perspective of a specific country?

- ◯ Global
- ◯ Country-specific

If you are responding with respect to a country different from the country where you currently work, please select the country.

[ ]

WHO Survey on Barriers to Implementation of PPH Interventions

WHO recommended intervention(s): Medicines/Devices
(page 1 of 10)

- **Each row represents a pre-requisite on the path to a successful implementation of WHO-recommended interventions for PPH.**
- **Please indicate the extent to which you agree or disagree with the statement in each row. Agreement indicates that the pre-requisite is in place and thus is NOT a barrier to implementation. Disagreement indicates that the pre-requisite is not in place and thus poses a barrier to implementation.**
- **Please score each row individually. You must score all rows to proceed to the next intervention.**
- **If you do not have an answer know for a particular pre-requisite, please tick "Don't know" in the relevant box.**

*** Oxytocin injection for PPH prevention and treatment**

	Strongly agree	Agree	Neither agree nor disagree	Disagree	Strongly disagree	Don't know
National guideline aligns and specifies how to deliver this intervention?	○	○	○	○	○	○
Stakeholders act as ‘champions’ to locally support implementation by raising awareness and encouraging uptake of this intervention?	○	○	○	○	○	○
Medicine is nationally registered and licensed for this indication?	○	○	○	○	○	○
Medicine included in national Essential Medicines List or national equivalent for this indication?	○	○	○	○	○	○
Medicine is available, and stock-outs are not an obstacle to use?	○	○	○	○	○	○
Appropriate job aids (e.g. clinical protocols) for this intervention are available at facility level?	○	○	○	○	○	○
Healthcare workers are aware and perceive this intervention as useful and effective?	○	○	○	○	○	○
Available products in the market are perceived as affordable and of good quality?	○	○	○	○	○	○
Healthcare workers are trained, experienced, and confident to consistently deliver this intervention?	○	○	○	○	○	○
Healthcare facilities are adequately staffed and equipped to consistently deliver this intervention?	○	○	○	○	○	○

Additional comments for the successful implementation of this intervention?

WHO Survey on Barriers to Implementation of PPH Interventions

WHO recommended intervention(s): Medicines/Devices

*** Ergometrine injection for PPH prevention and treatment (if oxytocin is unavailable)**

	Strongly agree	Agree	Neither agree not disagree	Disagree	Strongly disagree	Don't know
National guideline aligns and specifies how to deliver this intervention?	◯	◯	◯	◯	◯	◯
Stakeholders act as ‘champions’ to locally support implementation by raising awareness and encouraging uptake of this intervention?	◯	◯	◯	◯	◯	◯
Medicine is nationally registered and licensed for this indication?	◯	◯	◯	◯	◯	◯
Medicine included in national Essential Medicines List or national equivalent for this indication?	◯	◯	◯	◯	◯	◯
Medicine is available, and stock-outs are not an obstacle to use?	◯	◯	◯	◯	◯	◯
Appropriate job aids (e.g. clinical protocols) for this intervention are available at facility level?	◯	◯	◯	◯	◯	◯
Healthcare workers are aware and perceive this intervention as useful and effective?	◯	◯	◯	◯	◯	◯
Available products in the market are	◯	◯	◯	◯	◯	◯

perceived as affordable and of good quality?	○	○	○	○	○	○
Healthcare workers are trained, experienced, and confident to consistently deliver this intervention?	○	○	○	○	○	○
Healthcare facilities are adequately staffed and equipped to consistently deliver this intervention?	○	○	○	○	○	○

Additional comments for the successful implementation of this intervention?

[ ]

WHO Survey on Barriers to Implementation of PPH Interventions

WHO recommended intervention(s): Medicines/Devices (page 3 of 10)

*** Fixed-dose oxytocin and ergometrine combination injection for PPH prevention and treatment (if oxytocin is unavailable)**

	Strongly agree	Agree	Neither agree nor disagree	Disagree	Strongly disagree	Don't know
National guideline aligns and specifies how to deliver this intervention?	◯	◯	◯	◯	◯	◯
Stakeholders act as 'champions' to locally support implementation by raising awareness and encouraging uptake of this intervention?	◯	◯	◯	◯	◯	◯
Medicine is nationally registered and licensed for this indication?	◯	◯	◯	◯	◯	◯
Medicine included in national Essential Medicines List or national equivalent for this indication?	◯	◯	◯	◯	◯	◯
Medicine is available, and stock-outs are not an obstacle to use?	◯	◯	◯	◯	◯	◯
Appropriate job aids (e.g. clinical protocols) for this intervention are available at facility level?	◯	◯	◯	◯	◯	◯
Healthcare workers are aware and perceive this intervention as useful and effective?	◯	◯	◯	◯	◯	◯
Available products in the market are	◯	◯	◯	◯	◯	◯

perceived as affordable and of good quality?	○	○	○	○	○	○
Healthcare workers are trained, experienced, and confident to consistently deliver this intervention?	○	○	○	○	○	○
Healthcare facilities are adequately staffed and equipped to consistently deliver this intervention?	○	○	○	○	○	○

Additional comments for the successful implementation of this intervention?

WHO Survey on Barriers to Implementation of PPH Interventions

WHO recommended intervention(s): Medicines/Devices

*** Heat-stable carbetocin injection for PPH prevention (if oxytocin is unavailable or quality cannot be guaranteed)**

	Strongly agree	Agree	Neither agree nor disagree	Disagree	Strongly disagree	Don't know
National guideline aligns and specifies how to deliver this intervention?	○	○	○	○	○	○
Stakeholders act as ‘champions’ to locally support implementation by raising awareness and encouraging uptake of this intervention?	○	○	○	○	○	○
Medicine is nationally registered and licensed for this indication?	○	○	○	○	○	○
Medicine included in national Essential Medicines List or national equivalent for this indication?	○	○	○	○	○	○
Medicine is available, and stock-outs are not an obstacle to use?	○	○	○	○	○	○
Appropriate job aids (e.g. clinical protocols) for this intervention are available at facility level?	○	○	○	○	○	○
Healthcare workers are aware and perceive this intervention as useful and effective?	○	○	○	○	○	○
Available products in the market are	○	○	○	○	○	○

perceived as affordable and of good quality?	◯	◯	◯	◯	◯	◯
Healthcare workers are trained, experienced, and confident to consistently deliver this intervention?	◯	◯	◯	◯	◯	◯
Healthcare facilities are adequately staffed and equipped to consistently deliver this intervention?	◯	◯	◯	◯	◯	◯

Additional comments for the successful implementation of this intervention?

WHO recommended intervention(s): Medicines/Devices

*** Oral misoprostol for PPH prevention and treatment (if oxytocin is unavailable or did not stop the bleeding)**

	Strongly agree	Agree	Neither agree nor disagree	Disagree	Strongly disagree	Don't know
National guideline aligns and specifies how to deliver this intervention?	○	○	○	○	○	○
Stakeholders act as ‘champions’ to locally support implementation by raising awareness and encouraging uptake of this intervention?	○	○	○	○	○	○
Medicine is nationally registered and licensed for this indication?	○	○	○	○	○	○
Medicine included in national Essential Medicines List or national equivalent for this indication?	○	○	○	○	○	○
Medicine is available, and stock-outs are not an obstacle to use?	○	○	○	○	○	○
Appropriate job aids (e.g. clinical protocols) for this intervention are available at facility level?	○	○	○	○	○	○
Healthcare workers are aware and perceive this intervention as useful and effective?	○	○	○	○	○	○
Available products in the market are	○	○	○	○	○	○

perceived as affordable and of good quality?	○	○	○	○	○	○
Healthcare workers are trained, experienced, and confident to consistently deliver this intervention?	○	○	○	○	○	○
Healthcare facilities are adequately staffed and equipped to consistently deliver this intervention?	○	○	○	○	○	○

Additional comments for the successful implementation of this intervention?

WHO recommended intervention(s): Medicines/Devices
(page 6 of 10)

*** Isotonic crystalloids for fluid resuscitation of women with PPH**

	Strongly agree	Agree	Neither agree not disagree	Disagree	Strongly disagree	Don't know
National guideline aligns and specifies how to deliver this intervention?	○	○	○	○	○	○
Stakeholders act as ‘champions’ to locally support implementation by raising awareness and encouraging uptake of this intervention?	○	○	○	○	○	○
Medicine is nationally registered and licensed for this indication?	○	○	○	○	○	○
Medicine included in national Essential Medicines List or national equivalent for this indication?	○	○	○	○	○	○
Medicine is available, and stock-outs are not an obstacle to use?	○	○	○	○	○	○
Appropriate job aids (e.g. clinical protocols) for this intervention are available at facility level?	○	○	○	○	○	○
Healthcare workers are aware and perceive this intervention as useful and effective?	○	○	○	○	○	○
Available products in the market are perceived as affordable and of good quality?	○	○	○	○	○	○
Healthcare workers are trained, experienced, and confident to consistently deliver this intervention?	○	○	○	○	○	○
Healthcare facilities are adequately staffed and equipped to consistently deliver this intervention?	○	○	○	○	○	○

Additional comments for the successful implementation of this intervention?

WHO Survey on Barriers to Implementation of PPH Interventions

WHO recommended intervention(s): Medicines/Devices

*** Tranexamic acid (TXA) injection plus standard care for PPH treatment**

	Strongly agree	Agree	Neither agree nor disagree	Disagree	Agree	Don't know
National guideline aligns and specifies how to deliver this intervention?	◯	◯	◯	◯	◯	◯
Stakeholders act as ‘champions’ to locally support implementation by raising awareness and encouraging uptake of this intervention?	◯	◯	◯	◯	◯	◯
Medicine is nationally registered and licensed for this indication?	◯	◯	◯	◯	◯	◯
Medicine included in national Essential Medicines List or national equivalent for this indication?	◯	◯	◯	◯	◯	◯
Medicine is available, and stock-outs are not an obstacle to use?	◯	◯	◯	◯	◯	◯
Appropriate job aids (e.g. clinical protocols) for this intervention are available at facility level?	◯	◯	◯	◯	◯	◯
Healthcare workers are aware and perceive this intervention as useful and effective?	◯	◯	◯	◯	◯	◯
Available products in the market are perceived as affordable and of good quality?	◯	◯	◯	◯	◯	◯
Healthcare workers are trained, experienced, and confident to consistently deliver this intervention?	◯	◯	◯	◯	◯	◯
Healthcare facilities are adequately staffed and equipped to consistently deliver this intervention?	◯	◯	◯	◯	◯	◯

Additional comments for the successful implementation of this intervention?

WHO Survey on Barriers to Implementation of PPH Interventions

WHO recommended intervention(s): Medicines/Devices
(page 8 of 10)

*** Oxytocin in combination with controlled cord traction for retained placenta**

	Strongly agree	Agree	Neither agree nor disagree	Disagree	Strongly disagree	Don't know
National guideline aligns and specifies how to deliver this intervention?	○	○	○	○	○	○
Stakeholders act as ‘champions’ to locally support implementation by raising awareness and encouraging uptake of this intervention?	○	○	○	○	○	○
Medicine is nationally registered and licensed for this indication?	○	○	○	○	○	○
Medicine included in national Essential Medicines List or national equivalent for this indication?	○	○	○	○	○	○
Medicine is available, and stock-outs are not an obstacle to use?	○	○	○	○	○	○
Appropriate job aids (e.g. clinical protocols) for this intervention are available at facility level?	○	○	○	○	○	○
Healthcare workers are aware and perceive this intervention as useful and effective?	○	○	○	○	○	○
Available products in the market are perceived as affordable and of good quality?	○	○	○	○	○	○
Healthcare workers are trained, experienced, and confident to consistently deliver this intervention?	○	○	○	○	○	○
Healthcare facilities are adequately staffed and equipped to consistently deliver this intervention?	○	○	○	○	○	○

Additional comments for the successful implementation of this intervention?

WHO recommended intervention(s): Medicines/Devices

*** Uterine balloon tamponade (UBT) for refractory PPH treatment**

	Don't know	Strongly disagree	Disagree	Neither agree or disagree	Agree	Strongly Agree
National guideline aligns and specifies how to deliver this intervention?	◯	◯	◯	◯	◯	◯
Stakeholders act as 'champions' to locally support implementation by raising awareness and encouraging uptake of this intervention?	◯	◯	◯	◯	◯	◯
Device is nationally registered and licensed for this indication?	◯	◯	◯	◯	◯	◯
Device included in national List of Priority Medical Devices or national equivalent for this indication?	◯	◯	◯	◯	◯	◯
Device is available, and stock-outs are not an obstacle to use?	◯	◯	◯	◯	◯	◯
Appropriate job aids (e.g. clinical protocols) for this intervention are available at facility level?	◯	◯	◯	◯	◯	◯
Healthcare workers are aware and perceive this intervention as useful and effective?	◯	◯	◯	◯	◯	◯
Available products in the market are perceived as affordable and of good quality?	◯	◯	◯	◯	◯	◯
Healthcare workers are trained, experienced, and confident to consistently deliver this intervention?	◯	◯	◯	◯	◯	◯
Healthcare facilities are adequately staffed and equipped to consistently deliver this intervention?	◯	◯	◯	◯	◯	◯

Additional comments for the successful implementation of this intervention?

WHO Survey on Barriers to Implementation of PPH Interventions

*** Non-pneumatic anti-shock garment (NASG) as temporizing measure for definitive PPH care**

	Don't know	Strongly disagree	Disagree	Neither agree or disagree	Agree	Strongly Agree
Standard treatment guideline aligns and specifies how to deliver this intervention?	○	○	○	○	○	○
Stakeholders act as ‘champions’ to locally support implementation by raising awareness and encouraging uptake of this intervention?	○	○	○	○	○	○
Device is nationally registered and licensed for this indication?	○	○	○	○	○	○
Device included in national List of Priority Medical Devices or national equivalent for this indication?	○	○	○	○	○	○
Device is available, and stock-outs are not an obstacle to use?	○	○	○	○	○	○
Appropriate job aids (e.g. clinical protocols) for this intervention are available at facility level?	○	○	○	○	○	○
Healthcare workers are aware and perceive this intervention as useful and effective?	○	○	○	○	○	○

Available products in the market are perceived as affordable and of good quality?	○	○	○	○	○	○
Healthcare workers are trained, experienced, and confident to consistently deliver this intervention?	○	○	○	○	○	○
Healthcare facilities are adequately staffed and equipped to consistently deliver this intervention?	○	○	○	○	○	○

Additional comments for the successful implementation of this intervention?

WHO Survey on Barriers to Implementation of PPH Interventions

Saving your responses to the Medicines/Devices track

Thank you for providing your assessment of the barriers to implementation within the Medicines/Devices track. Your survey is not yet submitted.

Please select "Next" below to begin the Clinical and health system interventions track.

WHO Survey on Barriers to Implementation of PPH Interventions

WHO recommended intervention(s): Clinical and health system interventions (page 1 of 10)

- **Each row represents a pre-requisite on the path to a successful implementation of WHO-recommended interventions for PPH.**
- **Please indicate the extent to which you agree or disagree with the statement in each row. Agreement indicates that the pre-requisite is in place and thus is NOT a barrier to implementation. Disagreement indicates that the pre-requisite is not in place and thus poses a barrier to implementation.**
- **Please score each row individually. You must score all rows to proceed to the next intervention.**
- **If you do not have an answer know for a particular pre-requisite, please tick "Don't know" in the relevant box.**

*** Uterine artery embolization for refractory PPH treatment**

	Strongly agree	Agree	Neither agree nor disagree	Disagree	Strongly disagree	Don't know
National guideline aligns and specifies how to deliver this intervention?	◯	◯	◯	◯	◯	◯
Stakeholders act as ‘champions’ to locally support implementation by raising awareness and encouraging uptake of this intervention?	◯	◯	◯	◯	◯	◯
Appropriate job aids (e.g. clinical protocols) for this intervention are available at facility level?	◯	◯	◯	◯	◯	◯
Healthcare workers are aware and perceive this intervention as useful and effective?	◯	◯	◯	◯	◯	◯
Healthcare workers are trained, experienced, and confident to consistently deliver this intervention?	◯	◯	◯	◯	◯	◯
Healthcare facilities are adequately staffed and equipped to consistently deliver this intervention?	◯	◯	◯	◯	◯	◯

Additional comments for the successful implementation of this intervention?

WHO Survey on Barriers to Implementation of PPH Interventions

*** Bimanual uterine compression as temporizing measure before definitive PPH care**

	Strongly agree	Agree	Neither agree nor disagree	Disagree	Strongly disagree	Don't know
National guideline aligns and specifies how to deliver this intervention?	○	○	○	○	○	○
Stakeholders act as ‘champions’ to locally support implementation by raising awareness and encouraging uptake of this intervention?	○	○	○	○	○	○
Appropriate job aids (e.g. clinical protocols) for this intervention are available at facility level?	○	○	○	○	○	○
Healthcare workers are aware and perceive this intervention as useful and effective?	○	○	○	○	○	○
Healthcare workers are trained, experienced, and confident to consistently deliver this intervention?	○	○	○	○	○	○
Healthcare facilities are adequately staffed and equipped to consistently deliver this intervention?	○	○	○	○	○	○

Additional comments for the successful implementation of this intervention?

WHO recommended intervention(s): Clinical and health system interventions (page 3 of 10)

*** External aortic compression as temporizing measure for definitive PPH care**

	Strongly agree	Agree	Neither agree nor disagree	Disagree	Strongly disagree	Don't know
National guideline aligns and specifies how to deliver this intervention?	○	○	○	○	○	○
Stakeholders act as ‘champions’ to locally support implementation by raising awareness and encouraging uptake of this intervention?	○	○	○	○	○	○
Appropriate job aids (e.g. clinical protocols) for this intervention are available at facility level?	○	○	○	○	○	○
Healthcare workers are aware and perceive this intervention as useful and effective?	○	○	○	○	○	○
Healthcare workers are trained, experienced, and confident to consistently deliver this intervention?	○	○	○	○	○	○
Healthcare facilities are adequately staffed and equipped to consistently deliver this intervention?	○	○	○	○	○	○

Additional comments for the successful implementation of this intervention?

WHO recommended intervention(s): Clinical and health system interventions (page 4 of 10)

*** Surgical interventions (laparotomy with vessel ligation or compressive sutures or hysterectomy) for refractory PPH treatment**

	Strongly agree	Agree	Neither agree nor disagree	Disagree	Strongly disagree	Don't know
National guideline aligns and specifies how to deliver this intervention?	○	○	○	○	○	○
Stakeholders act as ‘champions’ to locally support implementation by raising awareness and encouraging uptake of this intervention?	○	○	○	○	○	○
Appropriate job aids (e.g. clinical protocols) for this intervention are available at facility level?	○	○	○	○	○	○
Healthcare workers are aware and perceive this intervention as useful and effective?	○	○	○	○	○	○
Healthcare workers are trained, experienced, and confident to consistently deliver this intervention?	○	○	○	○	○	○
Healthcare facilities are adequately staffed and equipped to consistently deliver this intervention?	○	○	○	○	○	○

Additional comments for the successful implementation of this intervention?

WHO Survey on Barriers to Implementation of PPH Interventions

WHO recommended intervention(s): Clinical and health system interventions (page 5 of 10)

*** Abdominal uterine tonus assessment for early identification of uterine atony for all women postpartum**

	Strongly agree	Agree	Neither agree nor disagree	Disagree	Strongly disagree	Don't know
National guideline aligns and specifies how to deliver this intervention?	○	○	○	○	○	○
Stakeholders act as ‘champions’ to locally support implementation by raising awareness and encouraging uptake of this intervention?	○	○	○	○	○	○
Appropriate job aids (e.g. clinical protocols) for this intervention are available at facility level?	○	○	○	○	○	○
Healthcare workers are aware and perceive this intervention as useful and effective?	○	○	○	○	○	○
Healthcare workers are trained, experienced, and confident to consistently deliver this intervention?	○	○	○	○	○	○
Healthcare facilities are adequately staffed and equipped to consistently deliver this intervention?	○	○	○	○	○	○

Additional comments for the successful implementation of this intervention?

WHO Survey on Barriers to Implementation of PPH Interventions

WHO recommended intervention(s): Clinical and health system interventions (page 6 of 10)

*** Controlled cord traction is the recommended method for removal of the placenta in caesarean section**

	Strongly agree	Agree	Neither agree not disagree	Disagree	Strongly disagree	Don't know
National guideline aligns and specifies how to deliver this intervention?	○	○	○	○	○	○
Stakeholders act as ‘champions’ to locally support implementation by raising awareness and encouraging uptake of this intervention?	○	○	○	○	○	○
Appropriate job aids (e.g. clinical protocols) for this intervention are available at facility level?	○	○	○	○	○	○
Healthcare workers are aware and perceive this intervention as useful and effective?	○	○	○	○	○	○
Healthcare workers are trained, experienced, and confident to consistently deliver this intervention?	○	○	○	○	○	○
Healthcare facilities are adequately staffed and equipped to consistently deliver this intervention?	○	○	○	○	○	○

Additional comments for the successful implementation of this intervention?

WHO Survey on Barriers to Implementation of PPH Interventions

WHO recommended intervention(s): Clinical and health system interventions (page 7 of 10)

*** Uterine massage for conservative treatment of PPH**

	Strongly agree	Agree	Neither agree nor disagree	Disagree	Strongly disagree	Don't know
National guideline aligns and specifies how to deliver this intervention?	○	○	○	○	○	○
Stakeholders act as ‘champions’ to locally support implementation by raising awareness and encouraging uptake of this intervention?	○	○	○	○	○	○
Appropriate job aids (e.g. clinical protocols) for this intervention are available at facility level?	○	○	○	○	○	○
Healthcare workers are aware and perceive this intervention as useful and effective?	○	○	○	○	○	○
Healthcare workers are trained, experienced, and confident to consistently deliver this intervention?	○	○	○	○	○	○
Healthcare facilities are adequately staffed and equipped to consistently deliver this intervention?	○	○	○	○	○	○

Additional comments for the successful implementation of this intervention?

WHO Survey on Barriers to Implementation of PPH Interventions

WHO recommended intervention(s): Clinical and health system interventions (page 8 of 10)

*** Formal protocols at health facilities for prevention and treatment of PPH**

	Strongly agree	Agree	Neither agree nor disagree	Disagree	Strongly disagree	Don't know
National guideline aligns and specifies how to deliver this intervention?	○	○	○	○	○	○
Stakeholders act as ‘champions’ to locally support implementation by raising awareness and encouraging uptake of this intervention?	○	○	○	○	○	○
Appropriate job aids (e.g. clinical protocols) for this intervention are available at facility level?	○	○	○	○	○	○
Healthcare workers are aware and perceive this intervention as useful and effective?	○	○	○	○	○	○
Healthcare workers are trained, experienced, and confident to consistently deliver this intervention?	○	○	○	○	○	○
Healthcare facilities are adequately staffed and equipped to consistently deliver this intervention?	○	○	○	○	○	○

Additional comments for the successful implementation of this intervention?

WHO Survey on Barriers to Implementation of PPH Interventions

WHO recommended intervention(s): Clinical and health system interventions (page 9 of 10)

*** Formal protocols for referral of women to a higher level of care for treatment of PPH**

	Strongly agree	Agree	Neither agree nor disagree	Disagree	Strongly disagree	Don't know
National guideline aligns and specifies how to deliver this intervention?	○	○	○	○	○	○
Stakeholders act as ‘champions’ to locally support implementation by raising awareness and encouraging uptake of this intervention?	○	○	○	○	○	○
Appropriate job aids (e.g. clinical protocols) for this intervention are available at facility level?	○	○	○	○	○	○
Healthcare workers are aware and perceive this intervention as useful and effective?	○	○	○	○	○	○
Healthcare workers are trained, experienced, and confident to consistently deliver this intervention?	○	○	○	○	○	○
Healthcare facilities are adequately staffed and equipped to consistently deliver this intervention?	○	○	○	○	○	○

Additional comments for the successful implementation of this intervention?

World Health Organization

human reproduction programme hrp
research for impact
UNDP·UNFPA·UNICEF·WHO·WORLD BANK

WHO recommended intervention(s): Clinical and health system interventions (page 10 of 10)

*** Simulations of PPH treatment for pre-service and in-service training programmes**

	Strongly agree	Agree	Neither agree nor disagree	Disagree	Strongly disagree	Don't know
National guideline aligns and specifies how to deliver this intervention?	○	○	○	○	○	○
Stakeholders act as ‘champions’ to locally support implementation by raising awareness and encouraging uptake of this intervention?	○	○	○	○	○	○
Appropriate job aids (e.g. clinical protocols) for this intervention are available at facility level?	○	○	○	○	○	○
Healthcare workers are aware and perceive this intervention as useful and effective?	○	○	○	○	○	○
Healthcare workers are trained, experienced, and confident to consistently deliver this intervention?	○	○	○	○	○	○
Healthcare facilities are adequately staffed and equipped to consistently deliver this intervention?	○	○	○	○	○	○

Additional comments for the successful implementation of this intervention?

WHO Survey on Barriers to Implementation of PPH Interventions

Saving your responses to the Clinical and health system interventions track

Thank you for providing your assessment of the barriers to implementation within the Clinical and health system interventions track. Your survey is not yet submitted.

Please select "Done" below to submit your responses.
