# Supplementary material for: Barriers and bottlenecks to implementation of WHO recommendations for prevention, detection, and treatment of postpartum hemorrhage: a mixed-methods triangulation study
Source: Implement Sci Commun. 2026 Jun 10;7:153. doi: 10.1186/s43058-026-00912-3 (PMC13483687; doi:10.1186/s43058-026-00912-3)
Supplement: Supplementary file 2 [file 43058_2026_912_MOESM2_ESM.pdf]

Nigeria health care worker responses

ID	Intervention	National guidelines align and specify how to deliver this intervention?	Stakeholders act as 'champions' to locally support implementation in countries by raising awareness and	Medicine/Device is registered and licensed for this indication?	Medicine / Device included in national Essential Medicines Lists or national equivalents for this indication?	Medicine/Device is available, and stock-outs are not an obstacle to use?	Appropriate job aids (e.g. clinical protocols) for this intervention are available at facility level?	Healthcare workers are aware and perceive this intervention as useful and effective?	Available products in the markets are perceived as affordable and of good quality?	Healthcare workers are trained, experienced, and confident to consistently deliver this intervention?	Healthcare facilities are adequately staffed and equipped to consistently
Q01	Oxytocin injection for PPH prevention and treatment	92%	85%	92%	86%	65%	76%	92%	59%	82%	56%
Q02	Ergometrine injection for PPH prevention and treatment (if oxytocin is unavailable)	79%	69%	85%	83%	60%	66%	75%	65%	71%	52%
Q03	Fixed-dose oxytocin and ergometrine combination injection for PPH prevention and treatment (if oxytocin is unavailable)	70%	64%	72%	70%	42%	59%	63%	52%	60%	44%
Q04	Heat-stable carbetocin injection for PPH prevention (if oxytocin is unavailable or quality cannot be guaranteed)	62%	63%	72%	71%	38%	43%	54%	48%	47%	46%
Q05	Oral misoprostol for PPH prevention and treatment (if oxytocin is unavailable or did not stop the bleeding)	94%	91%	90%	92%	80%	77%	87%	81%	84%	68%
Q06	Isotonic crystalloids for fluid resuscitation of women with PPH	88%	77%	86%	88%	66%	75%	86%	77%	78%	63%
Q07	Tranexamic acid (TXA) injection plus standard care for PPH treatment	74%	73%	77%	70%	55%	59%	68%	63%	63%	55%
Q08	Oxytocin in combination with controlled cord traction for retained placenta	90%	87%	90%	91%	75%	85%	90%	73%	78%	65%
Q09	Uterine balloon tamponade (UBT) for refractory PPH treatment	45%	49%	49%	45%	36%	36%	50%	37%	33%	29%
Q10	Non-pneumatic anti-shock garment (NASG) as temporizing measure for definitive PPH care	73%	69%	80%	79%	46%	63%	75%	51%	53%	50%
Q11	Uterine artery embolization for refractory PPH treatment	51%	44%				34%	56%		31%	28%
Q12	Bimanual uterine compression as temporizing measure before definitive PPH care	75%	67%				56%	72%		54%	49%
Q13	External aortic compression as temporizing measure for definitive PPH care	59%	50%				42%	52%		39%	37%
Q14	Surgical interventions (laparotomy with vessel ligation or compressive sutures or hysterectomy) for refractory PPH treatment	74%	70%				55%	73%		53%	46%
Q15	Abdominal uterine tonus assessment for early identification of uterine atony for all women postpartum	78%	77%				73%	79%		73%	64%
Q16	Controlled cord traction is the recommended method for removal of the placenta in caesarean section	74%	75%				72%	75%		71%	59%
Q17	Uterine massage for conservative treatment of PPH	89%	83%				74%	87%		84%	63%
Q18	Formal protocols at health facilities for prevention and treatment of PPH	80%	74%				66%	72%		67%	56%
Q19	Formal protocols for referral of women to a higher level of care for treatment of PPH	79%	79%				69%	77%		68%	56%
Q20	Simulations of PPH treatment for pre-service and in-service training programmes	67%	66%				55%	64%		58%	49%
	**Average cross interventions**	75%	71%	79%	78%	56%	62%	72%	61%	62%	52%

Pakistan health care worker responses

ID	Intervention	National guidelines align and specify how to deliver this intervention?	Stakeholders act as 'champions' to locally support implementation in countries by raising awareness and	Medicine/Device is registered and licensed for this indication?	Medicine / Device included in national Essential Medicines Lists or national equivalents for this indication?	Medicine/Device is available, and stock-outs are not an obstacle to use?	Appropriate job aids (e.g. clinical protocols) for this intervention are available at facility level?	Healthcare workers are aware and perceive this intervention as useful and effective?	Available products in the markets are perceived as affordable and of good quality?	Healthcare workers are trained, experienced, and confident to consistently deliver this intervention?	Healthcare facilities are adequately staffed and equipped to consistently
Q01	Oxytocin injection for PPH prevention and treatment	89%	84%	92%	90%	82%	86%	90%	84%	89%	82%
Q02	Ergometrine injection for PPH prevention and treatment (if oxytocin is unavailable)	51%	45%	50%	46%	32%	43%	51%	40%	49%	43%
Q03	Fixed-dose oxytocin and ergometrine combination injection for PPH prevention and treatment (if oxytocin is unavailable)	52%	50%	53%	51%	38%	48%	54%	43%	50%	47%
Q04	Heat-stable carbetocin injection for PPH prevention (if oxytocin is unavailable or quality cannot be guaranteed)	41%	43%	43%	41%	33%	35%	42%	37%	42%	35%
Q05	Oral misoprostol for PPH prevention and treatment (if oxytocin is unavailable or did not stop the bleeding)	89%	88%	88%	87%	85%	86%	90%	88%	88%	86%
Q06	Isotonic crystalloids for fluid resuscitation of women with PPH	82%	81%	82%	81%	78%	78%	82%	80%	81%	80%
Q07	Tranexamic acid (TXA) injection plus standard care for PPH treatment	87%	87%	87%	86%	86%	87%	88%	86%	85%	87%
Q08	Oxytocin in combination with controlled cord traction for retained placenta	88%	87%	89%	87%	84%	85%	87%	87%	87%	85%
Q09	Uterine balloon tamponade (UBT) for refractory PPH treatment	68%	67%	65%	63%	56%	58%	63%	57%	59%	55%
Q10	Non-pneumatic anti-shock garment (NASG) as temporizing measure for definitive PPH care	52%	53%	49%	48%	40%	44%	47%	44%	44%	44%
Q11	Uterine artery embolization for refractory PPH treatment	62%	59%				49%	56%		44%	43%
Q12	Bimanual uterine compression as temporizing measure before definitive PPH care	69%	68%				69%	69%		69%	68%
Q13	External aortic compression as temporizing measure for definitive PPH care	58%	55%				52%	55%		49%	50%
Q14	Surgical interventions (laparotomy with vessel ligation or compressive sutures or hysterectomy) for refractory PPH treatment	75%	76%				73%	75%		64%	63%
Q15	Abdominal uterine tonus assessment for early identification of uterine atony for all women postpartum	85%	84%				83%	84%		82%	82%
Q16	Controlled cord traction is the recommended method for removal of the placenta in caesarean section	81%	82%				80%	80%		80%	79%
Q17	Uterine massage for conservative treatment of PPH	88%	89%				87%	88%		88%	87%
Q18	Formal protocols at health facilities for prevention and treatment of PPH	83%	83%				82%	83%		82%	81%
Q19	Formal protocols for referral of women to a higher level of care for treatment of PPH	83%	81%				80%	82%		79%	79%
Q20	Simulations of PPH treatment for pre-service and in-service training programmes	73%	74%				74%	77%		75%	72%
	**Average cross interventions**	73%	72%	70%	68%	61%	69%	72%	65%	69%	67%

Tanzania health care worker responses

Intervention	National guidelines align and specify how to deliver this intervention?	Stakeholders act as 'champions' to locally support implementation in countries by raising awareness and	Medicine/Device is registered and licensed for this indication?	Medicine / Device included in national Essential Medicines Lists or national equivalents for this indication?	Medicine/Device is available, and stock-outs are not an obstacle to use?	Appropriate job aids (e.g. clinical protocols) for this intervention are available at facility level?	Healthcare workers are aware and perceive this intervention as useful and effective?	Available products in the markets are perceived as affordable and of good quality?	Healthcare workers are trained, experienced, and confident to consistently deliver this intervention?	Healthcare facilities are adequately staffed and equipped to consistently
Oxytocin injection for PPH prevention and treatment	97%	90%	96%	94%	76%	81%	91%	78%	79%	59%
Ergometrine injection for PPH prevention and treatment (if oxytocin is unavailable)	68%	66%	75%	71%	46%	59%	61%	60%	53%	46%
Fixed-dose oxytocin and ergometrine combination injection for PPH prevention and treatment (if oxytocin is unavailable)	57%	53%	58%	59%	45%	46%	47%	47%	44%	43%
Heat-stable carbetocin injection for PPH prevention (if oxytocin is unavailable or quality cannot be guaranteed)	43%	42%	44%	46%	34%	37%	37%	37%	30%	31%
Oral misoprostol for PPH prevention and treatment (if oxytocin is unavailable or did not stop the bleeding)	82%	76%	80%	80%	68%	75%	74%	68%	68%	60%
Isotonic crystalloids for fluid resuscitation of women with PPH	94%	90%	91%	90%	81%	84%	89%	82%	84%	73%
Tranexamic acid (TXA) injection plus standard care for PPH treatment	82%	82%	84%	80%	63%	67%	72%	69%	71%	67%
Oxytocin in combination with controlled cord traction for retained placenta	91%	88%	88%	87%	81%	83%	84%	83%	80%	68%
Uterine balloon tamponade (UBT) for refractory PPH treatment	58%	56%	62%	62%	45%	46%	44%	49%	41%	36%
Non-pneumatic anti-shock garment (NASG) as temporizing measure for definitive PPH care	55%	50%	58%	54%	35%	38%	41%	40%	36%	33%
Uterine artery embolization for refractory PPH treatment	49%	40%				42%	42%		36%	34%
Bimanual uterine compression as temporizing measure before definitive PPH care	82%	80%				79%	75%		71%	59%
External aortic compression as temporizing measure for definitive PPH care	71%	67%				61%	61%		54%	51%
Surgical interventions (laparotomy with vessel ligation or compressive sutures or hysterectomy) for refractory PPH treatment	81%	78%				73%	76%		66%	57%
Abdominal uterine tonus assessment for early identification of uterine atony for all women postpartum	81%	76%				72%	75%		73%	62%
Controlled cord traction is the recommended method for removal of the placenta in caesarean section	67%	68%				65%	69%		68%	56%
Uterine massage for conservative treatment of PPH	93%	90%				92%	90%		88%	75%
Formal protocols at health facilities for prevention and treatment of PPH	88%	88%				82%	84%		81%	68%
Formal protocols for referral of women to a higher level of care for treatment of PPH	85%	85%				83%	84%		80%	67%
Simulations of PPH treatment for pre-service and in-service training programmes	75%	74%				72%	75%		70%	66%
**Average cross interventions**	75%	72%	73%	72%	57%	67%	69%	61%	64%	56%
